# Median nerve impairment in leprosy: how does it differ from the classic carpal tunnel syndrome?

**DOI:** 10.1055/s-0044-1792092

**Published:** 2024-12-10

**Authors:** Pedro Henrique Sirotheau Corrêa Alves, Fernanda de Oliveira Cirino, Leonardo Peixoto Garcia, João Paulo Moreira Fernandes, Andrea De Martino Luppi, Douglas Eulálio Antunes, Raquel Campos Pereira, Wilson Marques Junior, Isabela Maria Bernardes Goulart, Diogo Fernandes dos Santos

**Affiliations:** 1 Universidade Federal de Uberlândia, Faculdade de Medicina, Hospital das Clínicas, Centro de Referência Nacional em Hanseníase e Dermatologia Sanitária, Uberlândia MG, Brazil.; 2 Universidade Federal de Uberlândia, Faculdade de Medicina, Programa de Pós-Graduação em Ciências da Saúde, Uberlândia MG, Brazil.; 3 Universidade de São Paulo, Faculdade de Medicina de Ribeirão Preto, Departamento de Neurologia, Divisão de Distúrbios Neuromusculares, Ribeirão Preto SP, Brazil.; 4 Instituto Nacional de Ciências e Tecnologia (INCT) em Medicins Trsnslacional, Ribeirão Preto SP, Brazil.

**Keywords:** Leprosy, Peripheral Nerves, Ultrasonography, *Mycobacterium leprae*, Electromyography, Hanseníase, Nervos Periféricos, Ultrassonografia, *Mycobacterium leprae*, Eletromiografia

## Abstract

**Background** Carpal tunnel syndrome (CTS) has already been described as a possible form of neural leprosy presentation. However, the median nerve can be involved in this neuropathy in proximal segments and, sometimes, with an asymmetric impairment of the digital branches.

**Objective** To detail the pattern of median nerve impairment through nerve conduction study (NCS) and ultrasound evaluation.

**Methods** This cross-sectional study comprises 15 primary neural leprosy (PNL) patients and 14 patients with CTS who underwent peripheral nerve ultrasonography and NCS evaluation.

**Results** From the total, 92.8% of patients with CTS and 80% with PNL had bilateral impairment of the median nerve, with 27 nerves in each group. Considering the cross-sectional area (CSA) of the tunnel (Mt) segment, 63% of the nerves in the CTS and 74.1% in the PNL groups were found to be thickened, with an average CSA of 13.4 ± 4.4 and 12.4 ± 4.6, respectively ( *p* = 0.18). The CSA of the proximal tunnel (Mpt) segment showed thickening in only 3.7% in the CTS group and 96.3% in the PNL ( *p* < 0.0001), with an average of 6.6 ± 1.3 and 17.0 ± 6.7, respectively ( *p* < 0.001). Finally, 88.9% of the nerves in the PNL group and only 7.4% in the CSA ( *p* < 0.0001) showed a reduction in conduction velocity in the distal forearm, with an average of 41.0 ± 6.3 and 53.2 ± 5.2, respectively ( *p* < 0.0001).

**Conclusion** The presence of neural thickening and demyelinating impairment in the segments proximal to the carpal tunnel favors the diagnosis of leprosy.

## Resumo

**Antecedentes** A síndrome do túnel do carpo (STC) já foi descrita como uma possível forma de apresentação da hanseníase neural. No entanto, o nervo mediano pode estar envolvido nesta neuropatia em segmentos proximais e, por vezes, com comprometimento assimétrico dos ramos digitais.

**Objetivo** Detalhar o padrão de comprometimento do nervo mediano através da avaliação eletroneuromiográfica e ultrassonográfica.

**Métodos** Este estudo transversal é composto por 15 pacientes com hanseníase neural primária (HNP) e 14 pacientes com STC que foram submetidos à avaliação ultrassonográfica e eletroneuromiográfica dos nervos periféricos.

**Resultados** Do total, 92,8% dos pacientes com STC e 80% com HNP apresentaram comprometimento bilateral do nervo mediano, com 27 nervos em cada grupo. Considerando a área de secção transversa (AST) do segmento do túnel (Mt), verificou-se que 63% dos nervos com STC e 74,1% com PNL estavam espessados, com AST média de 13,4 ± 4,4 e 12,4 ± 4,6, respetivamente ( *p* = 0,18). A AST do segmento do túnel proximal (Mpt) apresentou espessamento em apenas 3,7% no grupo STC e 96,3% no grupo HNP ( *p* < 0,0001), com uma média de 6,6 ± 1,3 e 17,0 ± 6,7, respetivamente ( *p* < 0,001). Por fim, 88,9% dos nervos do grupo PNL e apenas 7,4% do AST ( *p* < 0,0001) apresentaram redução da velocidade de condução no antebraço distal, com média de 41,0 ± 6,3 e 53,2 ± 5,2, respetivamente ( *p* < 0,0001).

**Conclusão** A presença de espessamento neural e comprometimento desmielinizante nos segmentos proximais ao túnel do carpo favorece o diagnóstico de hanseníase.

## INTRODUCTION

Leprosy is a public health problem and represents the primary infectious etiology of peripheral neuropathy worldwide. The involvement of peripheral nerves can be observed in all clinical forms of leprosy, with different forms of presentation, severity, and extent of involvement depending on the individual's immunological response. 1

Clinically, leprosy neuropathy presents predominantly sensory and asymmetrical impairment, configuring, in most cases, a pattern of asymmetric multiple mononeuropathy. Furthermore, the presence of neural thickening, although not an exclusive clinical sign of leprosy neuropathy, contributes significantly to diagnosis, especially in endemic regions. 2 3

Neural impairment in leprosy may be due to the direct effect of *Mycobacterium leprae* infection, causing harmful effects directly on Schwann cells, with consequent contact demyelination. Another possible mechanism of neural impairment is injuries resulting from the immune-mediated inflammatory process, comprising both the action of antibodies and the activation of cytotoxic T lymphocytes. Furthermore, neural impairment may result from edema and mechanical processes, as direct aggression and inflammatory conditions make the peripheral nerve more susceptible to compressive effects. 4 5 6

Carpal tunnel syndrome (CTS) is a common clinical condition caused by median nerve compression inside the carpal canal. It is an inelastic fibro-osseous tunnel defined by the carpal bones and the flexor retinaculum. Despite being common, the differential diagnosis can be complex, as some preexisting pathologies increase the possibility of compressive median nerve damage. 7

The most common systemic causes associated with CTS are diabetes mellitus, rheumatoid arthritis, and hypothyroidism. It can also appear during pregnancy or from hormonal abnormalities and, in some cases, be secondary to a traumatic wrist accident or fracture. Numerous peripheral neuropathies can favor impairment of the median nerve in the carpal tunnel, such as inflammatory neuropathies (chronic inflammatory demyelinating polyneuropathy and its variants) and hereditary (hereditary pressure-susceptible neuropathy, familial amyloid polyneuropathy, and some hereditary sensory-motor neuropathies). 7 8

The CTS has already been described as a possible form of neural leprosy presentation. However, the median nerve can be involved in this neuropathy in proximal segments and, sometimes, with an asymmetric impairment of the digital branches. 9 10 11 Furthermore, as a prevalent condition, it may not be directly related to infectious neuropathies. Therefore, this study aims to detail the pattern of the median nerve impairment through electroneuromyographic (ENMG) and ultrasonography (US) evaluation, favoring the differential diagnosis between classic CTS and leprosy neuropathy.

## METHODS

### Ethics statement

The Ethics Committee of the Clinics Hospital of Uberlândia Medical School approved the study (CAAE: 74917223.8.0000.5152), and all participants provided written informed consent.

### Type of study and subjects

This cross-sectional study comprises two groups, encompassing 15 leprosy patients (LPs) and 14 patients diagnosed with CTS. The first group was attended at the outpatient clinic of a national reference center of leprosy and enrolled by intentional sampling in the diagnosis.

As eligibility criteria for the group of LPs, participants should have a primary neural leprosy (PNL) diagnosis with clinical evidence of sensory or motor-sensory impairment in the territory corresponding to the median nerve. The diagnosis fulfilled the following criteria: clinical evidence of peripheral neuropathy associated with the absence of skin lesions and negative slit skin smear bacilloscopy, but with molecular and/or histopathological evidence of *M. leprae* infection on peripheral nerve biopsy. 2 3 All patients in this study's PNL group presented an asymmetric multiple mononeuropathy, with impairment of nerves other than the median.

The CTS patients were followed at an orthopedic outpatient clinic without personal and/or family history of leprosy, and with evidence of sensory or sensory-motor impairment in the territory corresponding to the median nerve. Both groups underwent peripheral nerve US and nerve conduction studies (NCS) evaluation by the same investigators. Only patients with at least a reduction in sensory CV in the NCS were included in the CTS group.

Patients who showed other possible etiologies of peripheral neuropathies were excluded, namely those with chronic alcoholism, diabetes mellitus, thyroid disease and other hormonal dysfunctions, malnutrition, hereditary neuropathy, hepatitis B or C, HIV, and/or rheumatic and autoimmune diseases.

### Ultrasonography

All patients underwent multisegmental US of the peripheral nerves, performed by a board-certified radiologist with experience in peripheral nerve imaging, using a 12 to 13 MHz linear transducer model LOGIC P6 PRO (GE Medical Systems, Milwaukee, WI, United States). The investigator who performed the US sessions was blinded to the clinical and laboratory characteristics of the LPs to avoid interference in US outcomes.

For the median nerve examination, the study participants' arms were positioned by their respective sides and in supination. The median nerve was scanned at the wrist in the carpal tunnel (Mt) at the level of the pisiform bone and 4 cm proximal to the carpal tunnel (Mpt).

The US beam was kept perpendicular to the nerve to approximate the most reliable value of the cross-sectional areas (CSAs). During the examination, CSAs were measured by freehand delimitation at the inner borders of the echogenic rims of the nerves using the electronic cursor. We calculated the absolute difference between measurements of each nerve at the tunnel and proximal to the tunnel point: Mt to Mpt index for the median nerve (ΔMtpt).

### Nerve conduction study

The NCSs were performed by a board-certified neurophysiologist with experience in peripheral neuropathy using a MEB 4200K (Nihon Kodhen Corp., Tokyo, Japan). For motor conduction studies, recording electrodes were placed over the abductor pollicis brevis (APB) muscle (lateral thenar eminence), and the reference electrode was placed distally over the first metacarpal phalangeal joint. The electrical pulse duration was usually 200 μs, with a current sufficient to achieve supramaximal stimulation. The nerves were stimulated at three points:

- The wrist (between the tendons to the flexor carpi radialis and palmaris longus at a distance of 8 cm from the recording electrode);
- The distal forearm (4 cm proximal to the wrist); and
- The antecubital fossa (over the brachial artery pulse).

The median motor palmar study was not conducted. The latency and amplitude of the compound muscle action potential (CMAP) for each stimulation site were measured. A motor conduction velocity (CV) was calculated after at least two sites, one distal and one proximal, had been stimulated (normal values being amplitude ≥ 4.0 mV; DML < 4.4 ms; CV ≥ 50.0 m/s).

For sensory conduction studies, the orthodromic technique was used. The active electrode was placed over the middle of the wrist between the tendons to the flexor carpi radialis and palmaris longus, and the reference electrode was placed 3 to 4 cm proximally. An electrical pulse of 200μs duration, with sufficient current to achieve supramaximal stimulation, was used. The median nerve was stimulated at the index and middle finger (digits 2 and 3) at a distance of 14 cm. The amplitude and CV of the sensory nerve action potential (SNAP) were measured (normal values peak-to-peak amplitude ≥ 10.0 μV; CV ≥ 50.0 m/s).

### Statistical analysis

Continuous and dichotomous variables were used to evaluate differences between groups. The Shapiro-Wilk test was used to verify data normality before applying parametric or nonparametric analyses. The Mann-Whitney test was carried out to compare differences in US parameters between groups. The binomial test was used to assess differences between two proportions. All statistical analyses were performed using the GraphPad Prism (San Diego, CA, USA) software, version 7.0, with an alpha error threshold of 5%.

## RESULTS

There were 29 patients included (CTS: 14; PNL: 15), with an average age of 42.9 ± 17.3 years and a predominance of females (55.2%; 16/29). Furthermore, 92.8% (13/14) of patients with CTS and 80.0% (12/15) with PNL had bilateral impairment of the median nerve, totaling 27 nerves affected in each group. All patients were symptomatic, presenting sensory symptoms, particularly hypoesthesia, paresthesia, and pain in the median nerve territory.

Considering the CSA of the Mt segment, 63.0% (17/27) of the nerves in the CTS and 74.1% (20/27) of the PNL groups were found to be thickened, with an average of 13.4 ± 4.4 and 12.4 ± 4.6, respectively ( *p* = 0.18). The CSA of the Mpt segment showed the presence of thickening in only 3.7% (1/27) in the CTS group and 96.3% (26/27) in the PNL group ( *p* < 0.0001), with an average of 6.6 ± 1.3 and 17.0 ± 6.7 respectively ( *p* < 0.001), as shown in Table 1 .

**Table 1:** Ultrasound and electroneuromyographic features of median nerve among patients with primary neural leprosy and carpal tunnel syndrome

Parameters		PNL N = 27	CTS N = 27	*p* -value
Ultrasound				
**CSA**	Tunnel	13.4 (± 4.4)	12.4 (± 4.6)	0.1858
	Proximal tunnel (4 cm)	17.0 (± 6.7)	6.6 (± 1.3)	< 0.0001
	**Tunnel – Proximal tunnel**	-3.6 (± 6.5)	5.7 (± 4.0)	< 0.0001
**Morphology**	Tunnel	100% (27/27)	40.7% (11/27)	0.0042
	Proximal tunnel (4 cm)	100% (27/27)	−	−
**Doppler**	Tunnel	3.7% (1/27)	7.4% (2/27)	0.5525
	Proximal tunnel (4 cm)	3.7% (1/27)	−	−
**Electroneuromyography**				
**Motor nerve conduction study**	Distal motor latency (ms)	3.6 (± 0.9)	4.2 (± 1.1)	0.0883
	Distal CMAP amplitude (mv)	7.5 (± 2.3)	8.6 (± 2.1)	0.0892
	Proximal CV (m/s)	52.2 (± 2.8)	53.4 (± 3.1)	0.2057
	Distal CV (m/s)	41.0 (± 6.3)	53.2 (± 5.2)	< 0.0001
	Proximal - distal CV (m/s)	10.9 (± 6.1)	0.2 (± 4.3)	< 0.0001
	F wave	29.7 (± 1.3)	27.5 (± 2.1)	< 0.0001
**Sensory nerve conduction study**	SNAP amplitude of digit 2 (μv)	8.3 (± 7.8)	50.8 (± 34.3)	< 0.0001
	SNAP amplitude of digit 3 (μv)	8.8 (± 8.5)	51.6 (± 34.7)	< 0.0001
	Digit 2–wrist CV (m/s)	44.5 (± 4.2)	43.0 (± 5.8)	0.3631
	Digit 3–wrist CV (m/s)	44.6 (± 4.3)	42.9 (± 5.8)	0.3336
	Palm–wrist CV (m/s)	43.2 (± 6.0)	39.7 (± 6.2)	0.1111

Abbreviations: CSA, cross sectional area; CMAPs, compound muscle action potentials; CTS, carpal tunnel syndrome; CV, conduction velocity; PNL, primary neural leprosy; SNAPs, sensory nerve action potentials.

All nerves evaluated in the PNL group lost the usual fascicular pattern in the Mt and Mpt segments. In contrast, in the CTS group, morphological changes were observed in only 40.7% (11/27) in the Mt segment and no nerve evaluated in the Mpt segment ( *p* < 0.001). Doppler evaluation did not reveal relevant abnormalities in any of the groups assessed. The ΔMtpt in the PNL group was −3.6 ± 6.5 and 5.7 ± 4.0 in the CTS ( *p* < 0.0001), as shown in Table 1 . A ΔMtpt ≥ 2.2 indicates the diagnosis of CTS with a sensitivity of 100% and specificity of 96.3%.

Regarding NCS evaluation, there were no differences in distal motor latency, compound muscle action potentials amplitudes, and conduction velocities in the proximal forearm segment in the motor conduction study. However, 88.9% (24/27) of the nerves in the PNL group and only 7.4% in the CSA (2/27) showed a reduction in conduction velocity in the distal forearm ( *p* < 0.0001), with an average of 41.0 ± 6.3) and 53.2 ± 5.2), respectively ( *p* < 0.0001). The difference in conduction velocity in the proximal segment of the forearm about the distal was 10.9 ± 6.1 in the PNL group and 0.2 ± 4.3 in the CTS group ( *p* < 0.0001). A difference in CV measurements (proximal–distal segment of the forearm ≥ 5.0 indicates the diagnosis of leprosy with a sensitivity of 94.7% and specificity of 92.6%. The F wave study showed a more significant prolongation of latencies in the PNL group ( *p* < 0.0001) ( Table 1 ).

The sensory conduction study showed no differences regarding CV in all techniques used. However, the evaluation of the sensory action potentials obtained in the index and middle fingers showed a significant reduction in amplitudes in the PNL group ( *p* < 0.0001) ( Table 1 ). Furthermore, in 14.8% (4/27) of the nerves evaluated, there was an asymmetry in the SNAP amplitudes comparing the index and middle fingers.

## DISCUSSION

The present study systematically evaluated the measurements of the median nerve using a multisegmental US and NCS. Several previous studies have already investigated the measurements of the nerves in leprosy neuropathy. However, the differential diagnosis of hypertrophic neuropathies is always a diagnostic challenge, especially in endemic areas. Both US and NCS are important tools for diagnosing peripheral nervous system diseases. Although these tests' objective is not an etiological diagnosis, they can be used in association with a clinical assessment for the correct diagnosis, contributing to the prevention of disabilities in leprosy neuropathy. 1 2 3 10 11 12 13 14 15 16 17 18

In our study, we noticed that patients in the leprosy group often present median nerve involvement in the carpal tunnel segment. However, given the high prevalence of CTS, it is impossible to define whether leprosy favors the onset of this condition. Therefore, it is important to note that the presence of neural thickening in isolation at the carpal tunnel level is not sufficient for the differential diagnosis and does not corroborate the diagnosis of leprosy neuropathy in the absence of proximal nerve involvement. 10 19 Even so, the neural thickening observed among the leprosy patients in our study strongly suggests an association between these two conditions.

**Figure 1:**
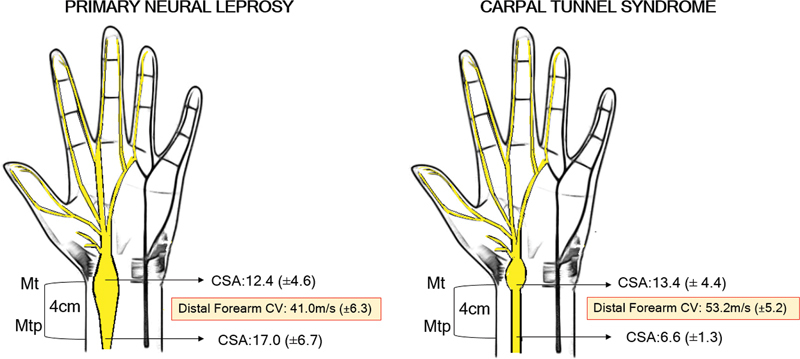
Comparison of neural thickening and demyelinating impairment of the median nerve in leprosy and carpal tunnel syndrome.

As observed, the presence of neural thickening and demyelinating involvement in the segments proximal to the carpal tunnel favors the diagnosis of leprosy ( Figure 1 ). This combination also corroborates the pathophysiology of the disease, in which the proliferation of the bacillus, especially in multibacillary forms, leads to a slow and progressive thickening of the peripheral nerve, with consequent contact demyelination. We can also observe greater axonal damage in some clinical forms depending on the host's immunological aggression and cellular immune response. 1 4 5 6

The leprosy neuropathy classically progresses with a temperature-dependent pattern of involvement. Therefore, the distal regions of the limbs may be involved, including asymmetries in the same neural trunk, as we observed in many cases. It is important to highlight that the observed asymmetry mainly reflects an axonal impairment, which differs from CTS. In this sense, a careful clinical evaluation is mandatory and corroborates the diagnosis of leprosy in the presence of the morphological or neurophysiological changes described. Furthermore, especially in cases of primarily neural leprosy, documenting the presence of the bacillus, even in peripheral nerve biopsy samples, represents a major challenge for early diagnosis. Therefore, new tools are always useful, especially when corroborating the patient's epidemiological and clinical context. 1 2 3 20 21 22

One of the benefits of morphological and neurophysiological assessment of the median nerve in patients with leprosy is the early approach to preventing disabilities. In classic CTS, surgical treatment is indicated in cases of moderate intensity, defined as motor impairment, confirmed by prolonged distal motor latencies. However, in many cases, individuals present evidence of demyelination proximal to the tunnel and sensory axonal degeneration, even with preserved distal motor latencies in ENMG. Therefore, considering that peripheral nerve's surgical decompression is also used as a complementary therapy to clinical neuritis treatment to preserve function, other measures must be considered in decision-making. 8 23

The median nerve is not one of the most affected in leprosy. However, when present, it contributes to the functional limitations imposed by the disease. This is the first study to evaluate the morphological and functional impairment of this nerve in a combined and systematic way, contributing to the prevention of disabilities and permanent sequelae in these individuals.

In conclusion, we propose the multisegmental US and NCS methods for evaluating the peripheral nerves when investigating the leprosy neuropathy. This analysis can help to discriminate leprosy from other neuropathy etiologies by revealing the asymmetry, irregular thickening, and demyelination, most evident above the osteofibrous tunnel of the nerves, which are characteristic of this condition.
